# Down-regulation of key regulatory factors in sphingosine-1-phosphate (S1P) pathway in human lung fibroblasts transfected with selected microRNAs

**DOI:** 10.22099/mbrc.2024.49810.1951

**Published:** 2024

**Authors:** Abdolamir Allameh, Mostafa Atashbasteh, Esmaeil Mortaz, Bahareh Naeeni, Majid Jafari-Khorchani

**Affiliations:** 1 Department of Clinical Biochemistry, Faculty of Medical Sciences, Tarbiat Modares University, Tehran, Iran; 2 Department of Immunology, Faculty of Medicine, Shahid Beheshti University of Medical Sciences, Tehran, Iran; 3 Clinical Tuberculosis and Epidemiology Research Center, National Research Institute of Tuberculosis and Lung Diseases, Shahid Beheshti University of Medical Sciences, Tehran, Iran

**Keywords:** Lung fibroblast, Regulation, micro RNAs: Sphingosine 1 phosphate, mRNA expression

## Abstract

Sphingosine 1 phosphate (S1P) is involved in the pathogenesis of asthma by stimulation of the alpha-smooth muscle actin (SMA) expression and remodeling of fibroblasts. This study was designed to determine the effects of selected micro RNAs in regulation of S1P and related metabolic pathways in a human lung fibroblast cell line. The fibroblast cell line (CIRC-HLF, C580) was cultured and transfected with individual viral vectors carrying miR124, mi125b, mi133b or mi130a. After 48 hours, expression level of miRNAs and their target genes, sphingosine kinase 1(SPHK1), sphingosine 1-phosphate lyase 1 (SGPL1), sphingosine 1- phosphate receptor 1 (S1PR1) and sphingosine 1- phosphate receptor 2 (S1PR2) were determined. Expression of miRNA and mRNA determined by reverse transcriptionquantitative polymerase chain reaction (qPCR) showed that the expression level of the miRNAs was significantly higher in human lung fibroblasts following transfection compared to controls (vector backbone without miRNA). The expressions of miRNAs-targeted genes were significantly downregulated in transfected fibroblasts compared to control cells (p<0.05). Data show that miR 124, miR 125b, miR 133b and miR130a by targeting regulatory genes in S1P-pathway can down-regulate key factors such as SPHK1, SGPL1, S1PR1 and S1PR2 genes in lung fibroblasts. The results showed that S1P pathway and key factors are suppressed in lung fibroblasts expressing miR124, miR125b, miR130a or miR133b. It appears that suppression of any of the intermediate factors in S1P by miRNA can affect the regulation of the entire S1P pathway.

## INTRODUCTION

Sphingosine -1 phosphate (S1P) is a metabolite of sphingolipid which is a potent bioactive lipid molecule involved in various cellular processes such as, regulation of immune system, cell growth, proliferation and apoptosis [1]. S1P is also known to promote the growth and proliferation of fibroblasts and production of extracellular matrix proteins which are associated with fibrotic lung remodeling [2, 3]. 

S1P regulates various processes such as cell motility and cytoskeletal rearrangements, survival, proliferation, angiogenesis and the trafficking of immune cells [4-6]. Ceramide (N-acyl sphingosine), plays a key role in sphingolipid metabolism and acts as a tumor-suppressor lipid, induce anti-proliferative and apoptotic responses in cancer cells. Evidences are also reported showing that S1P can induce responses which might render S1P a tumor-promoting factor[7].

In this pathway, sphingosine kinase types 1 and 2 (SphK1 and SphK2) to produce S1P phosphorylate sphingosine. In the case of the catabolism pathway, S1P is degraded either by reversible de-phosphorylation to sphingosine by phosphohydrolases or cleavage irreversibly to ethanolamine phosphate and hexadecenal by S1P lyase [4]. Pyne et al (2010), have reviewed the importance of S1P and S1P receptors (S1PR1-S1PR5) in the fate of cells, particularly cancer cells. According to this study, S1P regulates inflammatory reactions leading to induction of tumorigenesis; neovascularization, and increased cell growth and survival [8].

Information available in the literature on the role of S1P and receptors in lung fibroblasts is limited. Hashimoto et al, reported that S1P-augmented human lung fibroblast chemotaxis toward fibronectin relies on the S1P (2) receptor and requires Rho and Rho-kinase, and FAK phosphorylation. According to this study, by increasing fibroblast recruitment, S1P can participate in modulation of tissue repair after injury [9]. 

There are several factors, which are responsible for regulation of S1P metabolic pathways such as interleukins, hormones and growth factors [10-12]. More recently, regulation of S1P related pathways are facilitated by some microRNA (miRNAs) and long non-coding RNAs (Lnc-RNAs) [13-16]. It has been reported that some miRNAs and Lnc-RNAs play important role in allergic diseases and bronchial asthma [13]. miRNA, is a group of small non-coding RNAs, which can control the expression of target genes by suppression of translational process and mRNA degradation [17]. The interaction of miRNAs and the genes involved in S1P pathways has been reported from several laboratories. For example, miR-124 has been shown to inhibit cell proliferation through down-regulation of SPHK1 in gastric cancer [18]. Reported also show that miR-130a-3p regulates sphingosine1-phosphate receptor 2 (S1PR2) protein expression in human umbilical vein endothelial cells [19]. Likewise, miR-133b has been predicted to target S1PR1 which caused suppression of cell proliferation in nasopharyngeal carcinoma cells [20]. 

The part played by some miRNAs in inflammatory reactions has also been reported. For instance, miR-125b re-directly targets sphingosine-1-phosphate lyase 1(SGPL1) that leads to enhanced IL-8 production and the development of severe preeclampsia [21]. In case of asthma complication, miRNAs by targeting target genes particularly linked to S1P pathway can contribute to regulation of cell function and immune-inflammatory reactions in asthma development. In this line, recently we showed that among five miRNA molecules, miR-125b was overexpressed in exosomes derived from sera samples of patients suffering from severe asthma. However, other molecules; miR-124, miR-133b, and miR-130a were down-regulated in these patients [22].

The aim of the present study was to find out the possible interaction of the regulatory mRNAs with mRNA molecules related to S1P pathway in lung fibroblasts. For this purpose, expression of target genes involved in S1P pathway was determined in human lung fibroblasts following transfection with each of these mRNAs.

## MATERIALS AND METHODS

**Cell line and culture:** This experiment, was carried out using C580 (CIRC-HLF) cell line which is originated from human primary fibroblast cells isolated from lung biopsy of a diabetic woman. The cell line was obtained from the Cell Bank of the Pasteur Institute of Iran, Tehran, Iran. Cells were routinely cultured in Dulbecco’s Modified Eagle Medium (DMEM) (Cegrogen Biotech, Germany) supplemented with 10% fetal calf serum (Gibco, the USA) and Penicillin/Streptomycin1% (Bio-Idea, Tehran, Iran) in a humidified atmosphere of 5% CO2 at 37°C. To determine the target gene expression, first the CIRC-HLF cells were cultured in 6 well plate and then transfected with plasmids carrying individual miRNA. Transfection efficiency was estimated in terms of GFP expression, which was detected by florescence microscopy and flow cytometry. The rate of GFP-positive cells was checked after 24 and 48 hours and the rate of transfection in cells collected after 48 hours was >60%.

**Preparation of miRNA carrier vectors: **The lentiviruses containing each of the vectors carrying miRNAs and scramble (vector backbone without miRNA which is considered as negative control), were designed and synthesized by the Stem Cell Technology Research Center, Tehran, Iran. Vectors containing individual micro RNAs namely; miRNA124, miRNA125b and mi RNA133b were cloned in STBL4 strain of E. coli. DH5α strain of E. coli was used for cloning of micro RNA130a. The vectors also contained GFP (green fluorescent protein) gene, as the reporter gene with the potential to synthesize GFP protein. Each miRNA containing vector was first cloned in *Escherichia coli* (*E.* *coli*) and cultured on LB agar (LENNOX). After 24 h of colony formation, on Miller's LB medium, a single kanamycin -resistance colony was selected and cultured to the LB broth media and incubated for 24 hours as recommended by manufacturing protocols (Conda Pronadisa, Madrid, Spain). Thereafter, plasmids carrying miRNA were extracted from the E-coli using the FavorPrep plasmid extraction mini-kit (Favorgen, Taiwan) following the manufacturer’s instruction. The concentration of the extracted plasmids was determined on a NanoDrop (Thermo Fisher, USA). 

**Transfection of C580 (CIRC-HLF) fibroblast cells with Plenti-III-eGFP vector: **Transfection of the fibroblast cells was carried out using a transfection agent, Lipofectamine 2000 kit (Invitrogen, USA) with the lentiviral backbone plasmids (p-Lenti-III-eGFP-8852bp) following the manufacturer’s instruction. Briefly, transfection experiment was performed in 6-well culture plates. To each well approximately 4-8×10^5^ cells and 2 ml of incomplete medium (FBS- and antibiotic-free) was added. To increase the transfection efficiency, the cells were incubated at 37°C for 8-12 hours in a CO_2_ incubator. A solution containing 10 μl of lipofectamine 2000 and 240 μl of DMEM medium was prepared and allowed to stand at room temperature for 5 min. Then 4.5 μg of each of the vectors prepared in of DMEM medium (250 μl) was added to the reaction mixture and incubated at room temperature for 20 min. An aliquot (500 μl) of the reaction mixture was added to each well, incubated and checked at different time intervals to check the rate of transfection. The rate of the transfection was about 60% in the cells collected after 48 hours as shown by GFP expression determined by flow cytometry. Under this condition, the fibroblasts were separated for further analysis. 

**Extraction of total RNA from the fibroblasts transfected with miRNA vectors: **Total RNA was extracted from fibroblasts using Hybrid-RTM kit (GeneAll, South Korea) according to the procedure described by the manufacturer. The RNA concentration (ng/µL) and purity was quantified using NanoDrop-2000 spectrophotometer (Thermofisher technologies Inc., USA). 

In this experiment, the expression of miRNA124, miRNA 125b, miRNA133b and miRNA 130a were determined by reverse transcription technique using the BON-miR miRNA 1st-Strand kit (Stem Cell Technology Research Center, Tehran, Iran). This kit is working based on Poly (A)-Tailed universal Reverse Transcription method.

All the components were mixed and incubated for 30 min at 37°C before inactivation which was done for 20 min at 65°C for the polyadenylation reaction. Then to each tube, 10 μl of BON-RT adapter primer (10 µm) was added and the final volume was adjusted to 13 μl with RNase-free water before incubation at 75°C for 5 min. Finally, 1µl of RT enzyme, 2 µl of dNTP mix (100 mM) and 4 µl of 5X RT buffer were quickly added to each tube and the final volume was adjusted to 20 L. In case of mRNA, the IQ™ Supermix cDNA Synthesis kit (BioRad Lab. Inc., The USA) was used. Briefly, an aliquot (1 g) of mRNA sample was mixed with 4 L of reverse transcription mix and the final volume was adjusted to 20 L by adding DNase/RNase-free distilled water. The PCR protocol used as described below; 25C for 5 min and 42C for 30 min, and the reaction was stopped by a final step of 85C for 5 min.

**Quantitative reverse transcription PCR (qPCR):** QPCR was carried out using the BON-miR High- Specificity miRNA qPCR Kit (BON209002) for miRNA expression assay and BIOFACT SYBR Green PCR Master Mix (Including SYBR Green, BioFACT Co., Korea) for mRNA detection on an ABI 7500 real-time PCR system (Applied Biosystems, USA) following the instructions given by the manufacturer. The miRNA-specific primers were synthesized in Stem Cell Technology Research Center, Tehran, Iran (BON209001) and mRNA using primers was performed in Metabion (Germany). The miRNA expression was determined relative to the miR-U6, as housekeeping gene. Expression and mRNA cycle threshold (Ct) values were normalized to the GAPDH expression as an internal control. Finally results converted to fold change using the 2^-ΔΔCT^ formula by the Livak method [23].

**Statistical analysis: **QPCR analysis were performed in triplicate and analyzed by REST 2009 software, and the data presented are expressed as the mean ± SE using Graphpad Prism 8 Software. The differences between the groups were compared using Mann Whitney-U test for significance (p<0.05).

## Results

**Figure 1:**
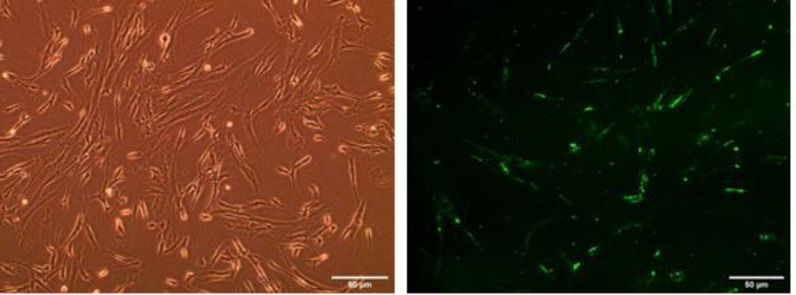
Transfection of the miRNA into human fibroblast C580 (CIRC-HLF) cell line. The fibroblasts before transfection (Left),and fibroblasts transfected with miRNA containing vectors (Right). The cells were examined under the fluorescence microscope (Olympus 1X, Japan). The magnification is 20X.

Transfection of the miRNA into the human fibroblsts was successfully carried out using GFP as reporter gene (Fig. 1).

**Figure 2:**
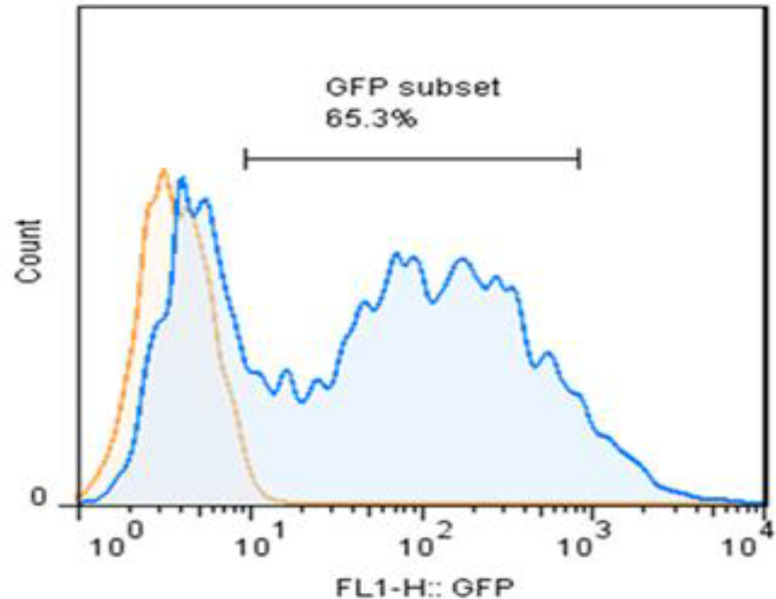
Characterization of the fibroblasts was performed using Flowcytometry analysis

Flow cytometry analysis was performed for further characterization of human lung fibroblasts infected with vector containing miR124. Green fluorescent protein expression was positive in more than 65% of the cells. The transfected cells were detected based on green fluorescent protein expression (Fig. 2). 

**Figure 3:**
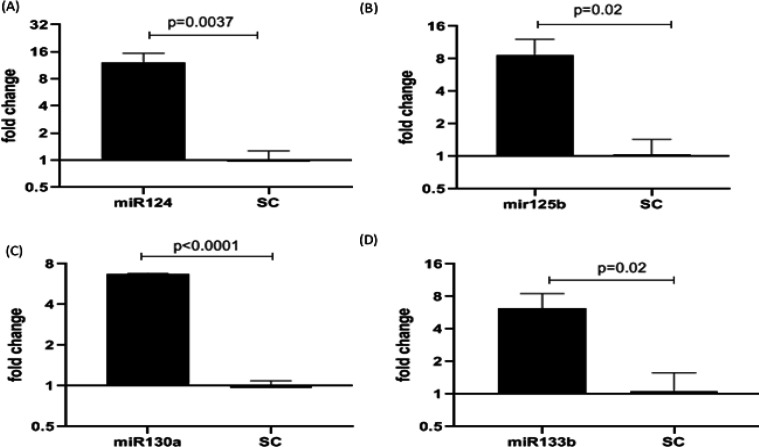
Overexpression of miRNA molecules in lung fibroblast following transfection. (A) miRNA 124, (B) miRNA 125b, (C) miRNA 133b and (D) miRNA 130a. Results are indicated as fold changes as expressed as Mean±SD of 3 samples carried out in duplicate. Details are as described under the methods section. Data are Mean±SD carried out in duplicate. (*p<0.05) is considered as statistically significant compared to cells received scramble (negative control).

The data presented in figure 3 show that the expression of the miR was significantly higher (6-12 folds) in the fibroblasts transfected with the vector containing miRNAs compared to the cells transfected with control (scrambles; vector backbone without miRNA). In this experiment, miRNA data were normalized to U6 as an internal control. Comparison of the expression of miRNA s in transfected cells with scramble (SC). 

**Figure 4:**
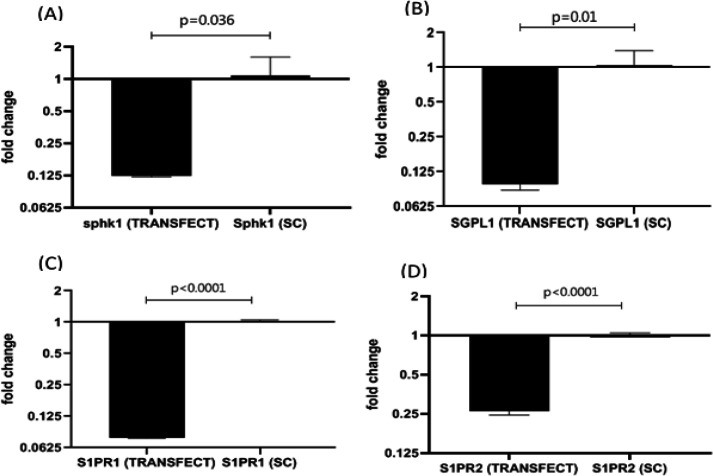
Expression level of target genes in lung fibroblast cells transfected with miRNAs. (A) Sphk1, target gene for miR124, (B) SGPL1, target gene for miR125b, (C) S1PR1, target gene for miR133b and S1PR2, target gene for miR130a. The data for miR containing vectors was compared with the negative control (scramble). Data are Mean±SD carried out in duplicate.

Expression of the mRNA molecules in the fibroblast cell line transfected with miRs containing vectors showed that the expression levels of SPHK1, SGPL1, S1PR1 and S1PR2 as the target genes in the cells transfected with, miR124, 125b, 130a or 133b was significantly down-regulated (Fig. 4). 

## DISCUSSION

The role of microRNAs (miRNA) in the regulation of cell signaling pathways is an emerging issue. The impact of the miRNAs on cell function and metabolism occurs mainly through regulation of gene expression. Evidences show that metabolism and function of the pulmonary fibroblasts could be affected by miRNAs under normal and disease condition. Therefore, the expression level of certain miRNAs as well their target genes in cells is important to understand the cellular and molecular basis of pulmonary diseases. For instance, it has been reported that progression of lung adenocarcinoma is associated with overexpression of miR-21 in cancer-associated fibroblasts (CAFs) [24].

Some miRNA molecules have been identified in relation to inflammatory reactions. Some of the miRNAs by targeting specific genes can cause modulation of inflammatory reactions. Lung fibroblasts, which are most abundant cell type in interstitium, play crucial role in airway inflammation and airway remodeling. Therefore, they are considered as target cells for treatment of inflammatory diseases such as asthma [25, 26]. S1P and its signaling pathway are well known for its involvement in the development of fibrosis, including pulmonary fibrosis, hepatic fibrosis, and cardiac fibrosis [27].

The impact of miRNA molecules on regulation of S1P enzymes and receptors could provide useful information in terms of molecular basis of fibroblast activities. This was evidenced by showing that certain miRNAs when transferred to lung fibroblasts could suppress the expression level of SPHK1, SGPL1, S1PR1, and S1PR2. Perhaps, suppression of key intermediate factors and receptors in S1P pathway can be considered as an important regulatory mechanism of fibroblast function and fate.

As a consequence of miRNA-mediated suppression of SPHK1, which is highly expressed in lungs, conversion of sphingosine to S1P is inhibited in fibroblasts expressing miRNAs [28]. Inhibition of the first step in S1P pathway can cause suppression in downstream intracellular pathways. Association of SGPL1 suppression with miRNAs was further confirmed by showing the suppression of SGPL1 following miRNA transfer into lung fibroblasts.

In our experiment, the fibroblasts transduced with miRNAs, in addition to SPHK1 and SGL1 enzymes, two of the major S1P receptors (S1PR1 and S1PR2) were also down-regulated. This finding clearly shows that S1P pathway is modulated in lung fibroblasts in presence of regulatory miRNA molecules. In this context, it appears that probably changes in a single key factor in initiation steps of the pathway can lead to control of S1P pathway and cellular responses.

Our experience on clinical samples showed that expression of plasma exosomal miR 124, miR125b, miR133b and miR 130a from patients suffering from asthma was associated with the inflammatory indices which are considered as diagnostic markers in patients suffering from severe asthma [22]. 

The relevance of S1P modulation by miRNAs and clinical conditions has been reported from different laboratories. The role of miR124 has been resembled to a tumor suppressor gene which is mediated by inhibition of SPHK1 gene in squamous cell carcinoma [29]. The impact of miR124 on S1P pathway has also been supported by showing that miR124-dependent regulation of SPHK1 on cell signaling pathways, AKT–FOXO1 [18]. 

In the present study, using lung fibroblast cell line, we could verify our previous findings, which was done on clinical samples of asthma showing the relationship of miRNAs to inflammatory reactions. As shown in figure 4, inhibition of SPHK1 expression in human lung fibroblast (HLF) cell line following transfection with the selected miRNA molecules, shows the involvement of these molecules in regulation of S1P pathway in lung fibroblasts. Our finding is in accordance with the report by Yang et al., (2016), which show that aberrant miRNA-125b expression is upregulated in basal and chorionic plates of preeclamptic placentas. This finding was further confirmed by showing that SGPL1 expression could be suppressed after transfection of miR-125b mimics in HTR8/SVneo cells. In contrast, overexpression of SGPL1 in trophoblast cells could reverse the IL-8 production enhancing effect of miR-125b expression [21].

In our experiments, the controlling role of miR-125b on target gene was approved by showing a negative relationship between the expressions of miR-125b with miR-125b containing vectors with the expression of the target gene, SGPL1 specific mRN**A** in transfected human lung fibroblast cell line.

Perhaps S1P regulation depends on binding of S1P to a family of five G protein-coupled receptors (S1PRs), known as S1P1–5[30]. The profile of S1P receptors can be changes in some cancers. as it was reported that the expression of S1P receptors, S1PR1, S1PR2 and S1PR3 is linked to the pathological grades and stages of bladder cancer [31]. Regulation of S1PR2 expression by miR-130a in human umbilical vein endothelial cells(HUVE) [19], indicates the contribution of miRs in S1P regulation, as well as it explains how these receptors are involved in S1P pathway. 

S1PR2 plays an vital role in the appearance and development of a various inflammatory diseases [32, 33]. According to Fan et al., TNFα could induce e inflammatory gene expression, which is likely mediated by the S1PR2 which suppress miR-130a-3p expression [19]. On the other hands, in the case of nasopharyngeal carcinoma (NPC) cells, endogenous S1PR1 protein expression was effectively modulated due to over-expression of miR 133b [20].

In conclusion, S1P pathway and related factors are suppressed in lung fibroblasts expressing miR124, miR125b, miR130a or miR133b. Hence, it appears that the regulation of entire S1P pathway will be disturbed in lung fibroblasts transduced with each of the miRNAs by suppressing SPHK1, SGPL1, S1PR1 or S1PR2. This finding is implicated in better understanding the role of miRNAs in regulation of S1P and signaling pathways related to inflammatory reactions.
